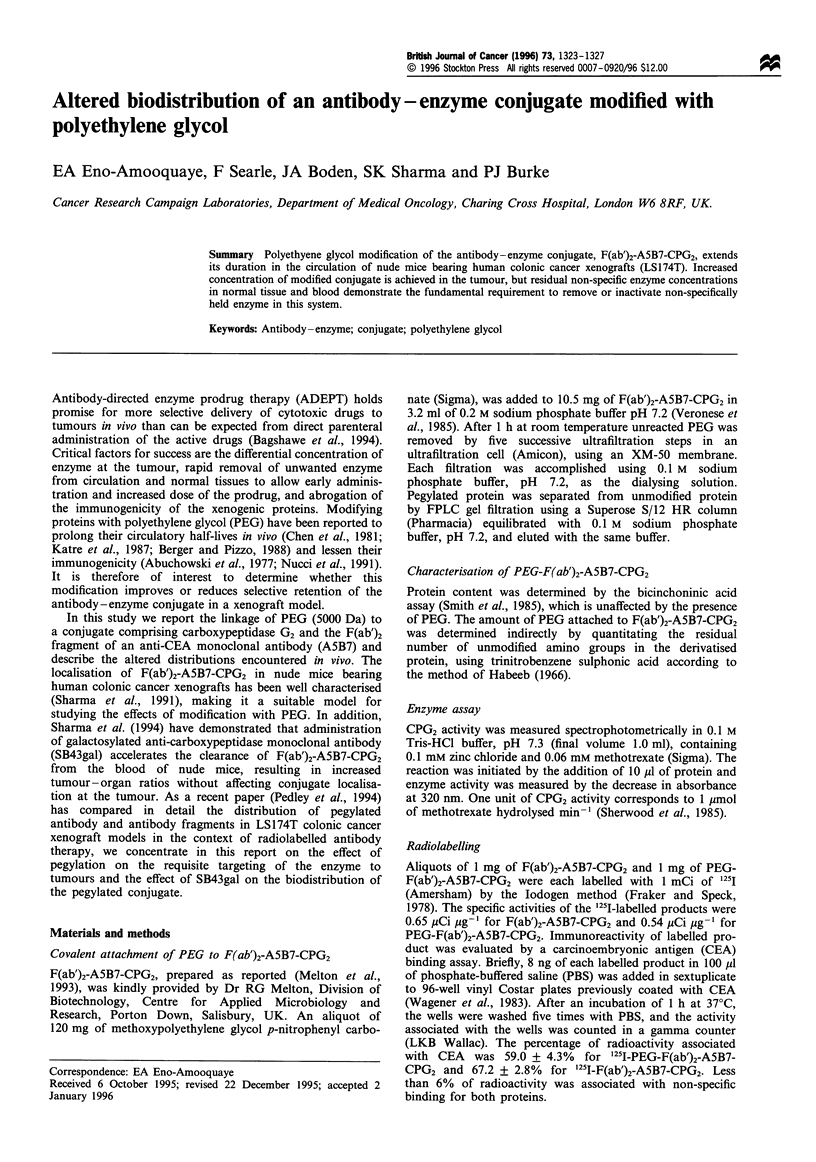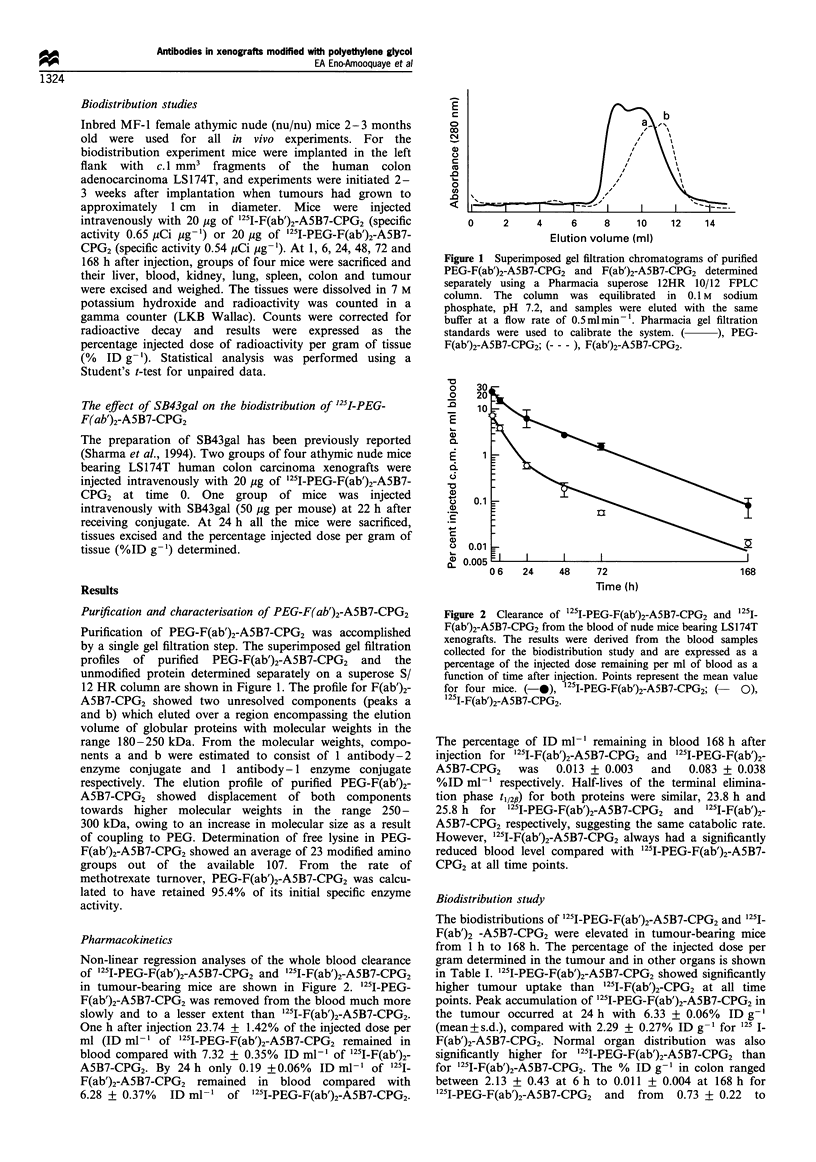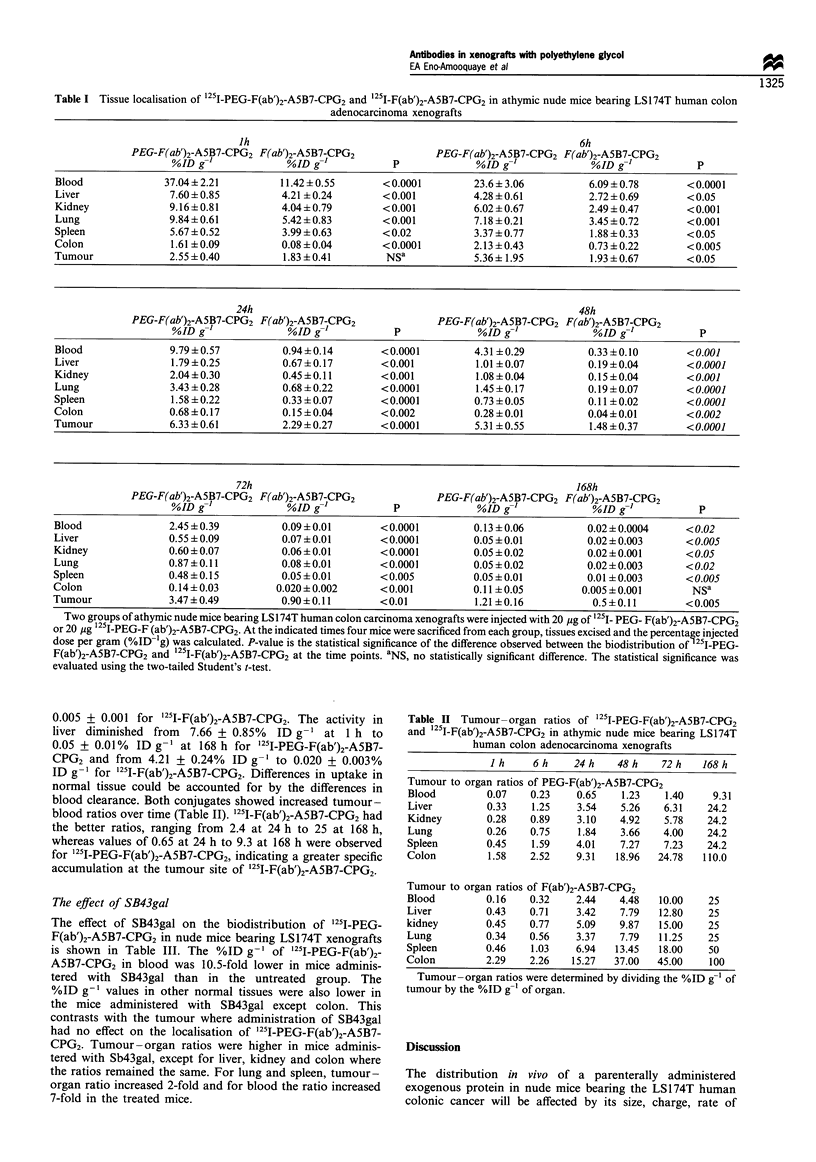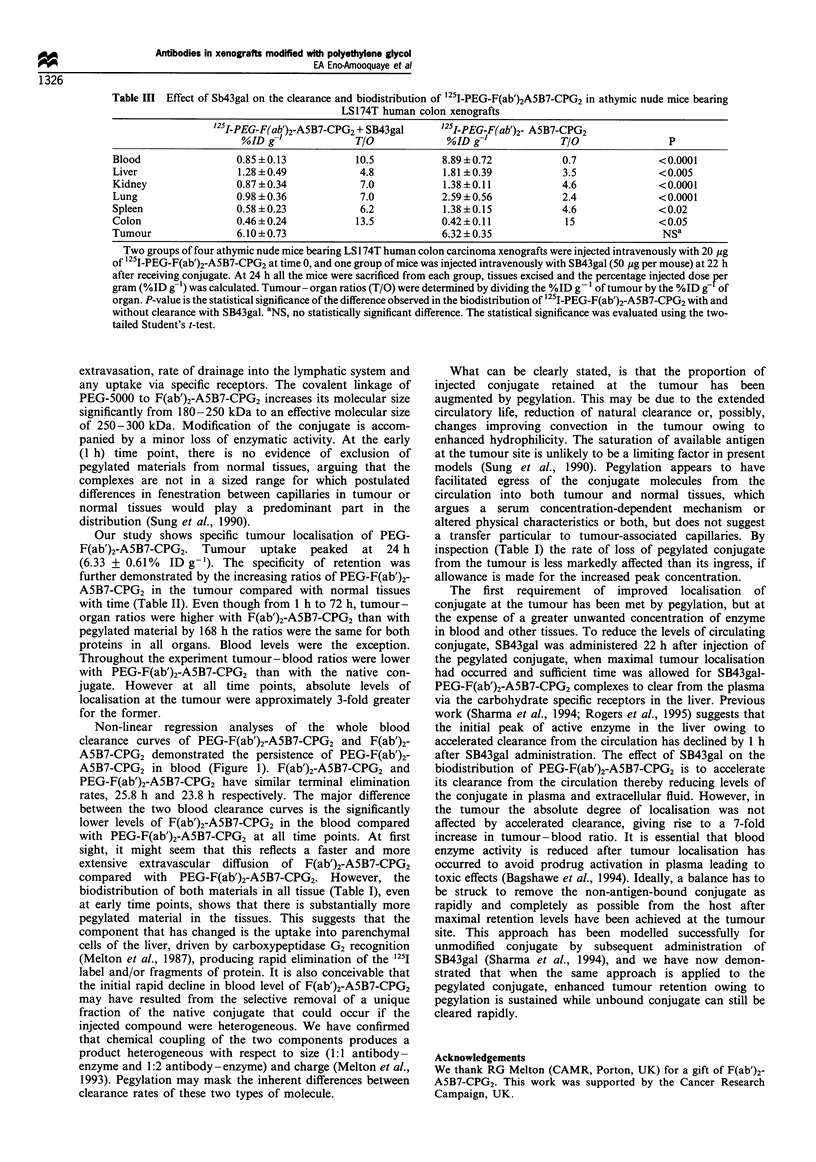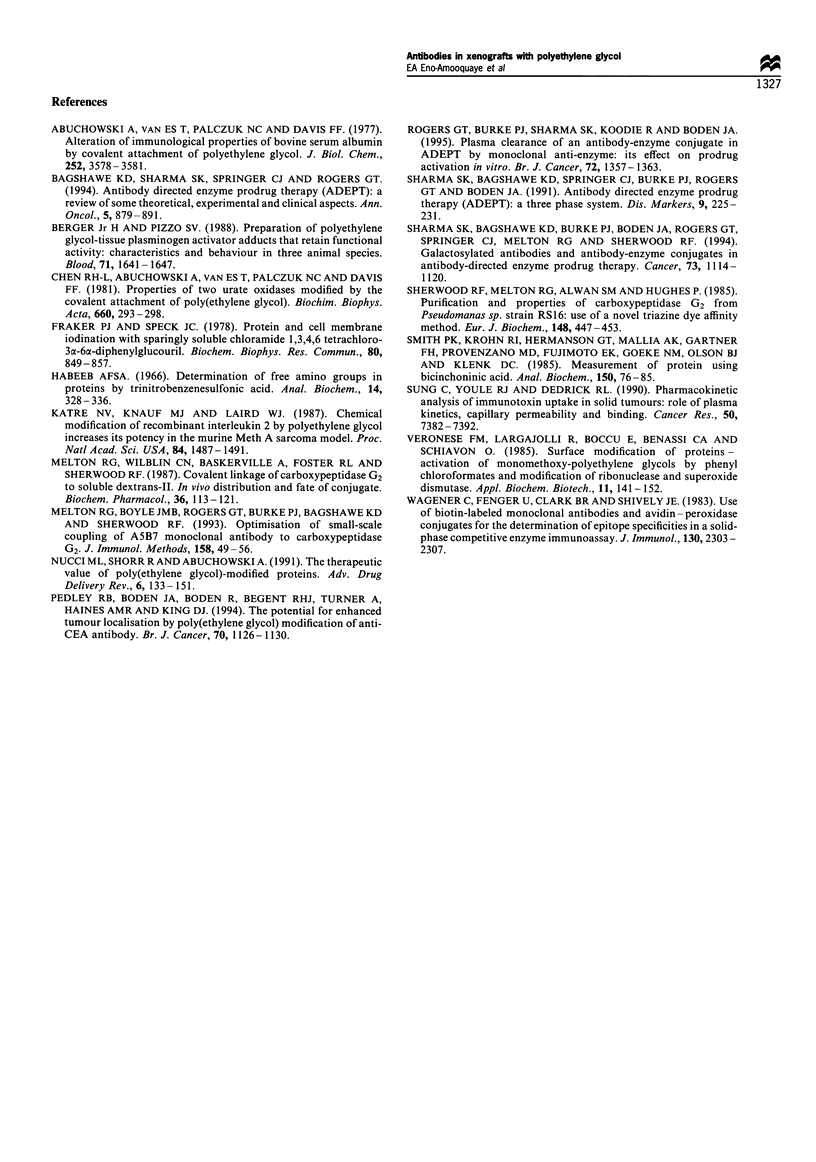# Altered biodistribution of an antibody--enzyme conjugate modified with polyethylene glycol.

**DOI:** 10.1038/bjc.1996.253

**Published:** 1996-06

**Authors:** E. A. Eno-Amooquaye, F. Searle, J. A. Boden, S. K. Sharma, P. J. Burke

**Affiliations:** Department of Medical Oncology, Charing Cross Hospital, London, UK.

## Abstract

Polyethylene glycol modification of the antibody--enzyme conjugate, F(ab')2-A5B7-CPG2, extends its duration in the circulation of nude mice bearing human colonic cancer xenografts (LS174T). Increased concentration of modified conjugate is achieved in the tumour, but residual non-specific enzyme concentrations in normal tissue and blood demonstrate the fundamental requirement to remove or inactivate non-specifically held enzyme in this system.


British Journal of Cancer (1996) 73, 1323-1327
© 1996 Stockton Press All rights reserved 0007-0920/96 $12.00

### Altered biodistribution of an antibody–enzyme conjugate modified with polyethylene glycol

EA Eno-Amooquaye, F Searle, JA Boden, SK Sharma and PJ Burke

*Cancer Research Campaign Laboratories, Department of Medical Oncology, Charing Cross Hospital, London W6 8RF, UK.*

**Summary** Polyethyene glycol modification of the antibody–enzyme conjugate, F(ab')$_2$-A5B7-CPG$_2$, extends its duration in the circulation of nude mice bearing human colonic cancer xenografts (LS174T). Increased concentration of modified conjugate is achieved in the tumour, but residual non-specific enzyme concentrations in normal tissue and blood demonstrate the fundamental requirement to remove or inactivate non-specifically held enzyme in this system.

**Keywords:** Antibody–enzyme; conjugate; polyethylene glycol

Antibody-directed enzyme prodrug therapy (ADEPT) holds promise for more selective delivery of cytotoxic drugs to tumours *in vivo* than can be expected from direct parenteral administration of the active drugs (Bagshawe *et al.*, 1994). Critical factors for success are the differential concentration of enzyme at the tumour, rapid removal of unwanted enzyme from circulation and normal tissues to allow early administration and increased dose of the prodrug, and abrogation of the immunogenicity of the xenogenic proteins. Modifying proteins with polyethylene glycol (PEG) have been reported to prolong their circulatory half-lives *in vivo* (Chen *et al.*, 1981; Katre *et al.*, 1987; Berger and Pizzo, 1988) and lessen their immunogenicity (Abuchowski *et al.*, 1977; Nucci *et al.*, 1991). It is therefore of interest to determine whether this modification improves or reduces selective retention of the antibody–enzyme conjugate in a xenograft model.

In this study we report the linkage of PEG (5000 Da) to a conjugate comprising carboxypeptidase G$_2$ and the F(ab')$_2$ fragment of an anti-CEA monoclonal antibody (A5B7) and describe the altered distributions encountered *in vivo*. The localisation of F(ab')$_2$-A5B7-CPG$_2$ in nude mice bearing human colonic cancer xenografts has been well characterised (Sharma *et al.*, 1991), making it a suitable model for studying the effects of modification with PEG. In addition, Sharma *et al.* (1994) have demonstrated that administration of galactosylated anti-carboxypeptidase monoclonal antibody (SB43gal) accelerates the clearance of F(ab')$_2$-A5B7-CPG$_2$ from the blood of nude mice, resulting in increased tumour–organ ratios without affecting conjugate localisation at the tumour. As a recent paper (Pedley *et al.*, 1994) has compared in detail the distribution of pegylated antibody and antibody fragments in LS174T colonic cancer xenograft models in the context of radiolabelled antibody therapy, we concentrate in this report on the effect of pegylation on the requisite targeting of the enzyme to tumours and the effect of SB43gal on the biodistribution of the pegylated conjugate.

### Materials and methods

### *Covalent attachment of PEG to F(ab')$_2$-A5B7-CPG$_2$*

F(ab')$_2$-A5B7-CPG$_2$, prepared as reported (Melton *et al.*, 1993), was kindly provided by Dr RG Melton, Division of Biotechnology, Centre for Applied Microbiology and Research, Porton Down, Salisbury, UK. An aliquot of 120 mg of methoxypolyethylene glycol *p*-nitrophenyl carbonate (Sigma), was added to 10.5 mg of F(ab')$_2$-A5B7-CPG$_2$ in 3.2 ml of 0.2 M sodium phosphate buffer pH 7.2 (Veronese *et al.*, 1985). After 1 h at room temperature unreacted PEG was removed by five successive ultrafiltration steps in an ultrafiltration cell (Amicon), using an XM-50 membrane. Each filtration was accomplished using 0.1 M sodium phosphate buffer, pH 7.2, as the dialysing solution. Pegylated protein was separated from unmodified protein by FPLC gel filtration using a Superose S/12 HR column (Pharmacia) equilibrated with 0.1 M sodium phosphate buffer, pH 7.2, and eluted with the same buffer.

### *Characterisation of PEG-F(ab')$_2$-A5B7-CPG$_2$*

Protein content was determined by the bicinchoninic acid assay (Smith *et al.*, 1985), which is unaffected by the presence of PEG. The amount of PEG attached to F(ab')$_2$-A5B7-CPG$_2$ was determined indirectly by quantitating the residual number of unmodified amino groups in the derivatised protein, using trinitrobenzene sulphonic acid according to the method of Habeeb (1966).

### *Enzyme assay*

CPG$_2$ activity was measured spectrophotometrically in 0.1 M Tris-HCl buffer, pH 7.3 (final volume 1.0 ml), containing 0.1 mM zinc chloride and 0.06 mM methotrexate (Sigma). The reaction was initiated by the addition of 10 $\mu$l of protein and enzyme activity was measured by the decrease in absorbance at 320 nm. One unit of CPG$_2$ activity corresponds to 1 $\mu$mol of methotrexate hydrolysed min$^{-1}$ (Sherwood *et al.*, 1985).

### *Radiolabelling*

Aliquots of 1 mg of F(ab')$_2$-A5B7-CPG$_2$ and 1 mg of PEG-F(ab')$_2$-A5B7-CPG$_2$ were each labelled with 1 mCi of $^{125}$I (Amersham) by the Iodogen method (Fraker and Speck, 1978). The specific activities of the $^{125}$I-labelled products were 0.65 $\mu$Ci $\mu$g$^{-1}$ for F(ab')$_2$-A5B7-CPG$_2$ and 0.54 $\mu$Ci $\mu$g$^{-1}$ for PEG-F(ab')$_2$-A5B7-CPG$_2$. Immunoreactivity of labelled product was evaluated by a carcinoembryonic antigen (CEA) binding assay. Briefly, 8 ng of each labelled product in 100 $\mu$l of phosphate-buffered saline (PBS) was added in sextuplicate to 96-well vinyl Costar plates previously coated with CEA (Wagener *et al.*, 1983). After an incubation of 1 h at 37°C, the wells were washed five times with PBS, and the activity associated with the wells was counted in a gamma counter (LKB Wallac). The percentage of radioactivity associated with CEA was 59.0 ± 4.3% for $^{125}$I-PEG-F(ab')$_2$-A5B7-CPG$_2$ and 67.2 ± 2.8% for $^{125}$I-F(ab')$_2$-A5B7-CPG$_2$. Less than 6% of radioactivity was associated with non-specific binding for both proteins.

Correspondence: EA Eno-Amooquaye
Received 6 October 1995; revised 22 December 1995; accepted 2 January 1996

*Biodistribution studies*

Inbred MF-1 female athymic nude (nu/nu) mice 2–3 months old were used for all *in vivo* experiments. For the biodistribution experiment mice were implanted in the left flank with *c*.1 $mm^3$ fragments of the human colon adenocarcinoma LS174T, and experiments were initiated 2–3 weeks after implantation when tumours had grown to approximately 1 cm in diameter. Mice were injected intravenously with 20 $\mu g$ of $^{125}$I-F(ab')$_2$-A5B7-CPG$_2$ (specific activity 0.65 $\mu Ci\ \mu g^{-1}$) or 20 $\mu g$ of $^{125}$I-PEG-F(ab')$_2$-A5B7-CPG$_2$ (specific activity 0.54 $\mu Ci\ \mu g^{-1}$). At 1, 6, 24, 48, 72 and 168 h after injection, groups of four mice were sacrificed and their liver, blood, kidney, lung, spleen, colon and tumour were excised and weighed. The tissues were dissolved in 7 M potassium hydroxide and radioactivity was counted in a gamma counter (LKB Wallac). Counts were corrected for radioactive decay and results were expressed as the percentage injected dose of radioactivity per gram of tissue (% ID $g^{-1}$). Statistical analysis was performed using a Student's *t*-test for unpaired data.

*The effect of SB43gal on the biodistribution of $^{125}$I-PEG-F(ab')$_2$-A5B7-CPG$_2$*

The preparation of SB43gal has been previously reported (Sharma *et al*., 1994). Two groups of four athymic nude mice bearing LS174T human colon carcinoma xenografts were injected intravenously with 20 $\mu g$ of $^{125}$I-PEG-F(ab')$_2$-A5B7-CPG$_2$ at time 0. One group of mice was injected intravenously with SB43gal (50 $\mu g$ per mouse) at 22 h after receiving conjugate. At 24 h all the mice were sacrificed, tissues excised and the percentage injected dose per gram of tissue (%ID $g^{-1}$) determined.

### Results

*Purification and characterisation of PEG-F(ab')$_2$-A5B7-CPG$_2$*

Purification of PEG-F(ab')$_2$-A5B7-CPG$_2$ was accomplished by a single gel filtration step. The superimposed gel filtration profiles of purified PEG-F(ab')$_2$-A5B7-CPG$_2$ and the unmodified protein determined separately on a superose S/12 HR column are shown in Figure 1. The profile for F(ab')$_2$-A5B7-CPG$_2$ showed two unresolved components (peaks a and b) which eluted over a region encompassing the elution volume of globular proteins with molecular weights in the range 180–250 kDa. From the molecular weights, components a and b were estimated to consist of 1 antibody–2 enzyme conjugate and 1 antibody–1 enzyme conjugate respectively. The elution profile of purified PEG-F(ab')$_2$-A5B7-CPG$_2$ showed displacement of both components towards higher molecular weights in the range 250–300 kDa, owing to an increase in molecular size as a result of coupling to PEG. Determination of free lysine in PEG-F(ab')$_2$-A5B7-CPG$_2$ showed an average of 23 modified amino groups out of the available 107. From the rate of methotrexate turnover, PEG-F(ab')$_2$-A5B7-CPG$_2$ was calculated to have retained 95.4% of its initial specific enzyme activity.

*Pharmacokinetics*

Non-linear regression analyses of the whole blood clearance of $^{125}$I-PEG-F(ab')$_2$-A5B7-CPG$_2$ and $^{125}$I-F(ab')$_2$-A5B7-CPG$_2$ in tumour-bearing mice are shown in Figure 2. $^{125}$I-PEG-F(ab')$_2$-A5B7-CPG$_2$ was removed from the blood much more slowly and to a lesser extent than $^{125}$I-F(ab')$_2$-A5B7-CPG$_2$. One h after injection 23.74 $\pm$ 1.42% of the injected dose per ml (ID $ml^{-1}$ of $^{125}$I-PEG-F(ab')$_2$-A5B7-CPG$_2$ remained in blood compared with 7.32 $\pm$ 0.35% ID $ml^{-1}$ of $^{125}$I-F(ab')$_2$-A5B7-CPG$_2$. By 24 h only 0.19 $\pm$0.06% ID $ml^{-1}$ of $^{125}$I-F(ab')$_2$-A5B7-CPG$_2$ remained in blood compared with 6.28 $\pm$ 0.37% ID $ml^{-1}$ of $^{125}$I-PEG-F(ab')$_2$-A5B7-CPG$_2$.

**Figure 1** Superimposed gel filtration chromatograms of purified PEG-F(ab')$_2$-A5B7-CPG$_2$ and F(ab')$_2$-A5B7-CPG$_2$ determined separately using a Pharmacia superose 12HR 10/12 FPLC column. The column was equilibrated in 0.1 M sodium phosphate, pH 7.2, and samples were eluted with the same buffer at a flow rate of 0.5 ml $min^{-1}$. Pharmacia gel filtration standards were used to calibrate the system. (———), PEG-F(ab')$_2$-A5B7-CPG$_2$; (- - - ), F(ab')$_2$-A5B7-CPG$_2$.

**Figure 2** Clearance of $^{125}$I-PEG-F(ab')$_2$-A5B7-CPG$_2$ and $^{125}$I-F(ab')$_2$-A5B7-CPG$_2$ from the blood of nude mice bearing LS174T xenografts. The results were derived from the blood samples collected for the biodistribution study and are expressed as a percentage of the injected dose remaining per ml of blood as a function of time after injection. Points represent the mean value for four mice. (—●), $^{125}$I-PEG-F(ab')$_2$-A5B7-CPG$_2$; (— ○), $^{125}$I-F(ab')$_2$-A5B7-CPG$_2$.

The percentage of ID $ml^{-1}$ remaining in blood 168 h after injection for $^{125}$I-F(ab')$_2$-A5B7-CPG$_2$ and $^{125}$I-PEG-F(ab')$_2$-A5B7-CPG$_2$ was 0.013 $\pm$ 0.003 and 0.083 $\pm$ 0.038 %ID $ml^{-1}$ respectively. Half-lives of the terminal elimination phase $t_{1/2\beta}$) for both proteins were similar, 23.8 h and 25.8 h for $^{125}$I-PEG-F(ab')$_2$-A5B7-CPG$_2$ and $^{125}$I-F(ab')$_2$-A5B7-CPG$_2$ respectively, suggesting the same catabolic rate. However, $^{125}$I-F(ab')$_2$-A5B7-CPG$_2$ always had a significantly reduced blood level compared with $^{125}$I-PEG-F(ab')$_2$-A5B7-CPG$_2$ at all time points.

*Biodistribution study*

The biodistributions of $^{125}$I-PEG-F(ab')$_2$-A5B7-CPG$_2$ and $^{125}$I-F(ab')$_2$ -A5B7-CPG$_2$ were elevated in tumour-bearing mice from 1 h to 168 h. The percentage of the injected dose per gram determined in the tumour and in other organs is shown in Table I. $^{125}$I-PEG-F(ab')$_2$-A5B7-CPG$_2$ showed significantly higher tumour uptake than $^{125}$I-F(ab')$_2$-CPG$_2$ at all time points. Peak accumulation of $^{125}$I-PEG-F(ab')$_2$-A5B7-CPG$_2$ in the tumour occurred at 24 h with 6.33 $\pm$ 0.06% ID $g^{-1}$ (mean$\pm$s.d.), compared with 2.29 $\pm$ 0.27% ID $g^{-1}$ for $^{125}$I-F(ab')$_2$-A5B7-CPG$_2$. Normal organ distribution was also significantly higher for $^{125}$I-PEG-F(ab')$_2$-A5B7-CPG$_2$ than for $^{125}$I-F(ab')$_2$-A5B7-CPG$_2$. The % ID $g^{-1}$ in colon ranged between 2.13 $\pm$ 0.43 at 6 h to 0.011 $\pm$ 0.004 at 168 h for $^{125}$I-PEG-F(ab')$_2$-A5B7-CPG$_2$ and from 0.73 $\pm$ 0.22 to

**Table I** Tissue localisation of $^{125}$I-PEG-F(ab')$_2$-A5B7-CPG$_2$ and $^{125}$I-F(ab')$_2$-A5B7-CPG$_2$ in athymic nude mice bearing LS174T human colon adenocarcinoma xenografts

| | 1h PEG-F(ab')$_2$-A5B7-CPG$_2$ %ID g$^{-1}$ | 1h F(ab')$_2$-A5B7-CPG$_2$ %ID g$^{-1}$ | P | 6h PEG-F(ab')$_2$-A5B7-CPG$_2$ %ID g$^{-1}$ | 6h F(ab')$_2$-A5B7-CPG$_2$ %ID g$^{-1}$ | P |
|---|---|---|---|---|---|---|
| Blood | 37.04±2.21 | 11.42±0.55 | <0.0001 | 23.6±3.06 | 6.09±0.78 | <0.0001 |
| Liver | 7.60±0.85 | 4.21±0.24 | <0.001 | 4.28±0.61 | 2.72±0.69 | <0.05 |
| Kidney | 9.16±0.81 | 4.04±0.79 | <0.001 | 6.02±0.67 | 2.49±0.47 | <0.001 |
| Lung | 9.84±0.61 | 5.42±0.83 | <0.001 | 7.18±0.21 | 3.45±0.72 | <0.001 |
| Spleen | 5.67±0.52 | 3.99±0.63 | <0.02 | 3.37±0.77 | 1.88±0.33 | <0.05 |
| Colon | 1.61±0.09 | 0.08±0.04 | <0.0001 | 2.13±0.43 | 0.73±0.22 | <0.005 |
| Tumour | 2.55±0.40 | 1.83±0.41 | NS[a] | 5.36±1.95 | 1.93±0.67 | <0.05 |

| | 24h PEG-F(ab')$_2$-A5B7-CPG$_2$ %ID g$^{-1}$ | 24h F(ab')$_2$-A5B7-CPG$_2$ %ID g$^{-1}$ | P | 48h PEG-F(ab')$_2$-A5B7-CPG$_2$ %ID g$^{-1}$ | 48h F(ab')$_2$-A5B7-CPG$_2$ %ID g$^{-1}$ | P |
|---|---|---|---|---|---|---|
| Blood | 9.79±0.57 | 0.94±0.14 | <0.0001 | 4.31±0.29 | 0.33±0.10 | *<0.001* |
| Liver | 1.79±0.25 | 0.67±0.17 | <0.001 | 1.01±0.07 | 0.19±0.04 | *<0.0001* |
| Kidney | 2.04±0.30 | 0.45±0.11 | <0.001 | 1.08±0.04 | 0.15±0.04 | *<0.001* |
| Lung | 3.43±0.28 | 0.68±0.22 | <0.0001 | 1.45±0.17 | 0.19±0.07 | *<0.0001* |
| Spleen | 1.58±0.22 | 0.33±0.07 | <0.0001 | 0.73±0.05 | 0.11±0.02 | *<0.0001* |
| Colon | 0.68±0.17 | 0.15±0.04 | <0.002 | 0.28±0.01 | 0.04±0.01 | *<0.002* |
| Tumour | 6.33±0.61 | 2.29±0.27 | <0.0001 | 5.31±0.55 | 1.48±0.37 | *<0.0001* |

| | 72h PEG-F(ab')$_2$-A5B7-CPG$_2$ %ID g$^{-1}$ | 72h F(ab')$_2$-A5B7-CPG$_2$ %ID g$^{-1}$ | P | 168h PEG-F(ab')$_2$-A5B7-CPG$_2$ %ID g$^{-1}$ | 168h F(ab')$_2$-A5B7-CPG$_2$ %ID g$^{-1}$ | P |
|---|---|---|---|---|---|---|
| Blood | 2.45±0.39 | 0.09±0.01 | <0.0001 | 0.13±0.06 | 0.02±0.0004 | *<0.02* |
| Liver | 0.55±0.09 | 0.07±0.01 | <0.0001 | 0.05±0.01 | 0.02±0.003 | *<0.005* |
| Kidney | 0.60±0.07 | 0.06±0.01 | <0.0001 | 0.05±0.02 | 0.02±0.001 | *<0.05* |
| Lung | 0.87±0.11 | 0.08±0.01 | <0.0001 | 0.05±0.02 | 0.02±0.003 | *<0.02* |
| Spleen | 0.48±0.15 | 0.05±0.01 | <0.005 | 0.05±0.01 | 0.01±0.003 | *<0.005* |
| Colon | 0.14±0.03 | 0.020±0.002 | <0.001 | 0.11±0.05 | 0.005±0.001 | NS[a] |
| Tumour | 3.47±0.49 | 0.90±0.11 | <0.01 | 1.21±0.16 | 0.5±0.11 | <0.005 |

Two groups of athymic nude mice bearing LS174T human colon carcinoma xenografts were injected with 20 $\mu$g of $^{125}$I- PEG- F(ab')$_2$-A5B7-CPG$_2$ or 20 $\mu$g $^{125}$I-PEG-F (ab')$_2$-A5B7-CPG$_2$. At the indicated times four mice were sacrificed from each group, tissues excised and the percentage injected dose per gram (%ID$^{-1}$g) was calculated. *P*-value is the statistical significance of the difference observed between the biodistribution of $^{125}$I-PEG-F(ab')$_2$-A5B7-CPG$_2$ and $^{125}$I-F(ab')$_2$-A5B7-CPG$_2$ at the time points. [a]NS, no statistically significant difference. The statistical significance was evaluated using the two-tailed Student's *t*-test.

0.005 ± 0.001 for $^{125}$I-F(ab')$_2$-A5B7-CPG$_2$. The activity in liver diminished from 7.66 ± 0.85% ID g$^{-1}$ at 1 h to 0.05 ± 0.01% ID g$^{-1}$ at 168 h for $^{125}$I-PEG-F(ab')$_2$-A5B7-CPG$_2$ and from 4.21 ± 0.24% ID g$^{-1}$ to 0.020 ± 0.003% ID g$^{-1}$ for $^{125}$I-F(ab')$_2$-A5B7-CPG$_2$. Differences in uptake in normal tissue could be accounted for by the differences in blood clearance. Both conjugates showed increased tumour–blood ratios over time (Table II). $^{125}$I-F(ab')$_2$-A5B7-CPG$_2$ had the better ratios, ranging from 2.4 at 24 h to 25 at 168 h, whereas values of 0.65 at 24 h to 9.3 at 168 h were observed for $^{125}$I-PEG-F(ab')$_2$-A5B7-CPG$_2$, indicating a greater specific accumulation at the tumour site of $^{125}$I-F(ab')$_2$-A5B7-CPG$_2$.

### *The effect of SB43gal*

The effect of SB43gal on the biodistribution of $^{125}$I-PEG-F(ab')$_2$-A5B7-CPG$_2$ in nude mice bearing LS174T xenografts is shown in Table III. The %ID g$^{-1}$ of $^{125}$I-PEG-F(ab')$_2$-A5B7-CPG$_2$ in blood was 10.5-fold lower in mice administered with SB43gal than in the untreated group. The %ID g$^{-1}$ values in other normal tissues were also lower in the mice administered with SB43gal except colon. This contrasts with the tumour where administration of SB43gal had no effect on the localisation of $^{125}$I-PEG-F(ab')$_2$-A5B7-CPG$_2$. Tumour–organ ratios were higher in mice administered with Sb43gal, except for liver, kidney and colon where the ratios remained the same. For lung and spleen, tumour–organ ratio increased 2-fold and for blood the ratio increased 7-fold in the treated mice.

**Table II** Tumour–organ ratios of $^{125}$I-PEG-F(ab')$_2$-A5B7-CPG$_2$ and $^{125}$I-F(ab')$_2$-A5B7-CPG$_2$ in athymic nude mice bearing LS174T human colon adenocarcinoma xenografts

| | *1 h* | *6 h* | *24 h* | *48 h* | *72 h* | *168 h* |
|---|---|---|---|---|---|---|
| Tumour to organ ratios of PEG-F(ab')$_2$-A5B7-CPG$_2$ | | | | | | |
| Blood | 0.07 | 0.23 | 0.65 | 1.23 | 1.40 | 9.31 |
| Liver | 0.33 | 1.25 | 3.54 | 5.26 | 6.31 | 24.2 |
| Kidney | 0.28 | 0.89 | 3.10 | 4.92 | 5.78 | 24.2 |
| Lung | 0.26 | 0.75 | 1.84 | 3.66 | 4.00 | 24.2 |
| Spleen | 0.45 | 1.59 | 4.01 | 7.27 | 7.23 | 24.2 |
| Colon | 1.58 | 2.52 | 9.31 | 18.96 | 24.78 | 110.0 |
| Tumour to organ ratios of F(ab')$_2$-A5B7-CPG$_2$ | | | | | | |
| Blood | 0.16 | 0.32 | 2.44 | 4.48 | 10.00 | 25 |
| Liver | 0.43 | 0.71 | 3.42 | 7.79 | 12.80 | 25 |
| kidney | 0.45 | 0.77 | 5.09 | 9.87 | 15.00 | 25 |
| Lung | 0.34 | 0.56 | 3.37 | 7.79 | 11.25 | 25 |
| Spleen | 0.46 | 1.03 | 6.94 | 13.45 | 18.00 | 50 |
| Colon | 2.29 | 2.26 | 15.27 | 37.00 | 45.00 | 100 |

Tumour–organ ratios were determined by dividing the %ID g$^{-1}$ of tumour by the %ID g$^{-1}$ of organ.

### Discussion

The distribution *in vivo* of a parenterally administered exogenous protein in nude mice bearing the LS174T human colonic cancer will be affected by its size, charge, rate of

Table III Effect of Sb43gal on the clearance and biodistribution of $^{125}$I-PEG-F(ab')$_2$A5B7-CPG$_2$ in athymic nude mice bearing LS174T human colon xenografts

| | $^{125}$I-PEG-F(ab')$_2$-A5B7-CPG$_2$ + SB43gal | | $^{125}$I-PEG-F(ab')$_2$- A5B7-CPG$_2$ | | |
|---|---|---|---|---|---|
| | %ID g$^{-1}$ | T/O | %ID g$^{-1}$ | T/O | P |
| Blood | 0.85 ± 0.13 | 10.5 | 8.89 ± 0.72 | 0.7 | <0.0001 |
| Liver | 1.28 ± 0.49 | 4.8 | 1.81 ± 0.39 | 3.5 | <0.005 |
| Kidney | 0.87 ± 0.34 | 7.0 | 1.38 ± 0.11 | 4.6 | <0.0001 |
| Lung | 0.98 ± 0.36 | 7.0 | 2.59 ± 0.56 | 2.4 | <0.0001 |
| Spleen | 0.58 ± 0.23 | 6.2 | 1.38 ± 0.15 | 4.6 | <0.02 |
| Colon | 0.46 ± 0.24 | 13.5 | 0.42 ± 0.11 | 15 | <0.05 |
| Tumour | 6.10 ± 0.73 | | 6.32 ± 0.35 | | NS[a] |

Two groups of four athymic nude mice bearing LS174T human colon carcinoma xenografts were injected intravenously with 20 μg of $^{125}$I-PEG-F(ab')$_2$-A5B7-CPG$_2$ at time 0, and one group of mice was injected intravenously with SB43gal (50 μg per mouse) at 22 h after receiving conjugate. At 24 h all the mice were sacrificed from each group, tissues excised and the percentage injected dose per gram (%ID g$^{-1}$) was calculated. Tumour–organ ratios (T/O) were determined by dividing the %ID g$^{-1}$ of tumour by the %ID g$^{-1}$ of organ. P-value is the statistical significance of the difference observed in the biodistribution of $^{125}$I-PEG-F(ab')$_2$-A5B7-CPG$_2$ with and without clearance with SB43gal. [a]NS, no statistically significant difference. The statistical significance was evaluated using the two-tailed Student's t-test.

extravasation, rate of drainage into the lymphatic system and any uptake via specific receptors. The covalent linkage of PEG-5000 to F(ab')$_2$-A5B7-CPG$_2$ increases its molecular size significantly from 180–250 kDa to an effective molecular size of 250–300 kDa. Modification of the conjugate is accompanied by a minor loss of enzymatic activity. At the early (1 h) time point, there is no evidence of exclusion of pegylated materials from normal tissues, arguing that the complexes are not in a sized range for which postulated differences in fenestration between capillaries in tumour or normal tissues would play a predominant part in the distribution (Sung *et al.*, 1990).

Our study shows specific tumour localisation of PEG-F(ab')$_2$-A5B7-CPG$_2$. Tumour uptake peaked at 24 h (6.33 ± 0.61% ID g$^{-1}$). The specificity of retention was further demonstrated by the increasing ratios of PEG-F(ab')$_2$-A5B7-CPG$_2$ in the tumour compared with normal tissues with time (Table II). Even though from 1 h to 72 h, tumour–organ ratios were higher with F(ab')$_2$-A5B7-CPG$_2$ than with pegylated material by 168 h the ratios were the same for both proteins in all organs. Blood levels were the exception. Throughout the experiment tumour–blood ratios were lower with PEG-F(ab')$_2$-A5B7-CPG$_2$ than with the native conjugate. However at all time points, absolute levels of localisation at the tumour were approximately 3-fold greater for the former.

Non-linear regression analyses of the whole blood clearance curves of PEG-F(ab')$_2$-A5B7-CPG$_2$ and F(ab')$_2$-A5B7-CPG$_2$ demonstrated the persistence of PEG-F(ab')$_2$-A5B7-CPG$_2$ in blood (Figure 1). F(ab')$_2$-A5B7-CPG$_2$ and PEG-F(ab')$_2$-A5B7-CPG$_2$ have similar terminal elimination rates, 25.8 h and 23.8 h respectively. The major difference between the two blood clearance curves is the significantly lower levels of F(ab')$_2$-A5B7-CPG$_2$ in the blood compared with PEG-F(ab')$_2$-A5B7-CPG$_2$ at all time points. At first sight, it might seem that this reflects a faster and more extensive extravascular diffusion of F(ab')$_2$-A5B7-CPG$_2$ compared with PEG-F(ab')$_2$-A5B7-CPG$_2$. However, the biodistribution of both materials in all tissue (Table I), even at early time points, shows that there is substantially more pegylated material in the tissues. This suggests that the component that has changed is the uptake into parenchymal cells of the liver, driven by carboxypeptidase G$_2$ recognition (Melton *et al.*, 1987), producing rapid elimination of the $^{125}$I label and/or fragments of protein. It is also conceivable that the initial rapid decline in blood level of F(ab')$_2$-A5B7-CPG$_2$ may have resulted from the selective removal of a unique fraction of the native conjugate that could occur if the injected compound were heterogeneous. We have confirmed that chemical coupling of the two components produces a product heterogeneous with respect to size (1:1 antibody–enzyme and 1:2 antibody–enzyme) and charge (Melton *et al.*, 1993). Pegylation may mask the inherent differences between clearance rates of these two types of molecule.

What can be clearly stated, is that the proportion of injected conjugate retained at the tumour has been augmented by pegylation. This may be due to the extended circulatory life, reduction of natural clearance or, possibly, changes improving convection in the tumour owing to enhanced hydrophilicity. The saturation of available antigen at the tumour site is unlikely to be a limiting factor in present models (Sung *et al.*, 1990). Pegylation appears to have facilitated egress of the conjugate molecules from the circulation into both tumour and normal tissues, which argues a serum concentration-dependent mechanism or altered physical characteristics or both, but does not suggest a transfer particular to tumour-associated capillaries. By inspection (Table I) the rate of loss of pegylated conjugate from the tumour is less markedly affected than its ingress, if allowance is made for the increased peak concentration.

The first requirement of improved localisation of conjugate at the tumour has been met by pegylation, but at the expense of a greater unwanted concentration of enzyme in blood and other tissues. To reduce the levels of circulating conjugate, SB43gal was administered 22 h after injection of the pegylated conjugate, when maximal tumour localisation had occurred and sufficient time was allowed for SB43gal-PEG-F(ab')$_2$-A5B7-CPG$_2$ complexes to clear from the plasma via the carbohydrate specific receptors in the liver. Previous work (Sharma *et al.*, 1994; Rogers *et al.*, 1995) suggests that the initial peak of active enzyme in the liver owing to accelerated clearance from the circulation has declined by 1 h after SB43gal administration. The effect of SB43gal on the biodistribution of PEG-F(ab')$_2$-A5B7-CPG$_2$ is to accelerate its clearance from the circulation thereby reducing levels of the conjugate in plasma and extracellular fluid. However, in the tumour the absolute degree of localisation was not affected by accelerated clearance, giving rise to a 7-fold increase in tumour–blood ratio. It is essential that blood enzyme activity is reduced after tumour localisation has occurred to avoid prodrug activation in plasma leading to toxic effects (Bagshawe *et al.*, 1994). Ideally, a balance has to be struck to remove the non-antigen-bound conjugate as rapidly and completely as possible from the host after maximal retention levels have been achieved at the tumour site. This approach has been modelled successfully for unmodified conjugate by subsequent administration of SB43gal (Sharma *et al.*, 1994), and we have now demonstrated that when the same approach is applied to the pegylated conjugate, enhanced tumour retention owing to pegylation is sustained while unbound conjugate can still be cleared rapidly.

**Acknowledgements**

We thank RG Melton (CAMR, Porton, UK) for a gift of F(ab')$_2$-A5B7-CPG$_2$. This work was supported by the Cancer Research Campaign, UK.

### References

ABUCHOWSKI A, VAN ES T, PALCZUK NC AND DAVIS FF. (1977). Alteration of immunological properties of bovine serum albumin by covalent attachment of polyethylene glycol. *J. Biol. Chem.*, **252,** 3578–3581.

BAGSHAWE KD, SHARMA SK, SPRINGER CJ AND ROGERS GT. (1994). Antibody directed enzyme prodrug therapy (ADEPT): a review of some theoretical, experimental and clinical aspects. *Ann. Oncol.*, **5,** 879–891.

BERGER Jr H AND PIZZO SV. (1988). Preparation of polyethylene glycol-tissue plasminogen activator adducts that retain functional activity: characteristics and behaviour in three animal species. *Blood*, **71,** 1641–1647.

CHEN RH-L, ABUCHOWSKI A, VAN ES T, PALCZUK NC AND DAVIS FF. (1981). Properties of two urate oxidases modified by the covalent attachment of poly(ethylene glycol). *Biochim. Biophys. Acta*, **660,** 293–298.

FRAKER PJ AND SPECK JC. (1978). Protein and cell membrane iodination with sparingly soluble chloramide 1,3,4,6 tetrachloro-3α-6α-diphenylglucouril. *Biochem. Biophys. Res. Commun.*, **80,** 849–857.

HABEEB AFSA. (1966). Determination of free amino groups in proteins by trinitrobenzenesulfonic acid. *Anal. Biochem.*, **14,** 328–336.

KATRE NV, KNAUF MJ AND LAIRD WJ. (1987). Chemical modification of recombinant interleukin 2 by polyethylene glycol increases its potency in the murine Meth A sarcoma model. *Proc. Natl Acad. Sci. USA*, **84,** 1487–1491.

MELTON RG, WILBLIN CN, BASKERVILLE A, FOSTER RL AND SHERWOOD RF. (1987). Covalent linkage of carboxypeptidase $G_2$ to soluble dextrans-II. *In vivo* distribution and fate of conjugate. *Biochem. Pharmacol.*, **36,** 113–121.

MELTON RG, BOYLE JMB, ROGERS GT, BURKE PJ, BAGSHAWE KD AND SHERWOOD RF. (1993). Optimisation of small-scale coupling of A5B7 monoclonal antibody to carboxypeptidase $G_2$. *J. Immunol. Methods*, **158,** 49–56.

NUCCI ML, SHORR R AND ABUCHOWSKI A. (1991). The therapeutic value of poly(ethylene glycol)-modified proteins. *Adv. Drug Delivery Rev.*, **6,** 133–151.

PEDLEY RB, BODEN JA, BODEN R, BEGENT RHJ, TURNER A, HAINES AMR AND KING DJ. (1994). The potential for enhanced tumour localisation by poly(ethylene glycol) modification of anti-CEA antibody. *Br. J. Cancer*, **70,** 1126–1130.

ROGERS GT, BURKE PJ, SHARMA SK, KOODIE R AND BODEN JA. (1995). Plasma clearance of an antibody-enzyme conjugate in ADEPT by monoclonal anti-enzyme: its effect on prodrug activation *in vitro*. *Br. J. Cancer*, **72,** 1357–1363.

SHARMA SK, BAGSHAWE KD, SPRINGER CJ, BURKE PJ, ROGERS GT AND BODEN JA. (1991). Antibody directed enzyme prodrug therapy (ADEPT): a three phase system. *Dis. Markers*, **9,** 225–231.

SHARMA SK, BAGSHAWE KD, BURKE PJ, BODEN JA, ROGERS GT, SPRINGER CJ, MELTON RG AND SHERWOOD RF. (1994). Galactosylated antibodies and antibody-enzyme conjugates in antibody-directed enzyme prodrug therapy. *Cancer*, **73,** 1114–1120.

SHERWOOD RF, MELTON RG, ALWAN SM AND HUGHES P. (1985). Purification and properties of carboxypeptidase $G_2$ from *Pseudomanas sp.* strain RS16: use of a novel triazine dye affinity method. *Eur. J. Biochem.*, **148,** 447–453.

SMITH PK, KROHN RI, HERMANSON GT, MALLIA AK, GARTNER FH, PROVENZANO MD, FUJIMOTO EK, GOEKE NM, OLSON BJ AND KLENK DC. (1985). Measurement of protein using bicinchoninic acid. *Anal. Biochem.*, **150,** 76–85.

SUNG C, YOULE RJ AND DEDRICK RL. (1990). Pharmacokinetic analysis of immunotoxin uptake in solid tumours: role of plasma kinetics, capillary permeability and binding. *Cancer Res.*, **50,** 7382–7392.

VERONESE FM, LARGAJOLLI R, BOCCU E, BENASSI CA AND SCHIAVON O. (1985). Surface modification of proteins – activation of monomethoxy-polyethylene glycols by phenyl chloroformates and modification of ribonuclease and superoxide dismutase. *Appl. Biochem. Biotech.*, **11,** 141–152.

WAGENER C, FENGER U, CLARK BR AND SHIVELY JE. (1983). Use of biotin-labeled monoclonal antibodies and avidin–peroxidase conjugates for the determination of epitope specificities in a solid-phase competitive enzyme immunoassay. *J. Immunol.*, **130,** 2303–2307.